# A Survey of Techniques for Discovering, Using, and Paying for Third-Party IoT Sensors

**DOI:** 10.3390/s24082539

**Published:** 2024-04-15

**Authors:** Anas Dawod, Dimitrios Georgakopoulos, Prem Prakash Jayaraman, Ampalavanapillai Nirmalathas

**Affiliations:** 1 Department of Computing Technologies, Swinburne University of Technology, Melbourne 3122, Australia; dgeorgakopoulos@swin.edu.au (D.G.); pjayaraman@swin.edu.au (P.P.J.); 2 Department of Electrical and Electronic Engineering, The University of Melbourne, Melbourne 3010, Australia; nirmalat@unimelb.edu.au

**Keywords:** cost-sharing, description, discovery, Internet of Things (IoT), integration, IoT device, payment, query, sensor, sharing

## Abstract

The Internet of Things (IoT) includes billions of sensors and actuators (which we refer to as IoT devices) that harvest data from the physical world and send it via the Internet to IoT applications to provide smart IoT services and products. Deploying, managing, and maintaining IoT devices for the exclusive use of an individual IoT application is inefficient and involves significant costs and effort that often outweigh the benefits. On the other hand, enabling large numbers of IoT applications to share available third-party IoT devices, which are deployed and maintained independently by a variety of IoT device providers, reduces IoT application development costs, time, and effort. To achieve a positive cost/benefit ratio, there is a need to support the sharing of third-party IoT devices globally by providing effective IoT device discovery, use, and pay between IoT applications and third-party IoT devices. A solution for global IoT device sharing must be the following: (1) scalable to support a vast number of third-party IoT devices, (2) interoperable to deal with the heterogeneity of IoT devices and their data, and (3) IoT-owned, i.e., not owned by a specific individual or organization. This paper surveys existing techniques that support discovering, using, and paying for third-party IoT devices. To ensure that this survey is comprehensive, this paper presents our methodology, which is inspired by Systematic Literature Network Analysis (SLNA), combining the Systematic Literature Review (SLR) methodology with Citation Network Analysis (CNA). Finally, this paper outlines the research gaps and directions for novel research to realize global IoT device sharing.

## 1. Introduction

The Internet of Things (IoT) combines billions of Internet-accessible IoT devices (e.g., sensors, RFIDs, wearables, smartphones, smart meters, industrial machines, vehicles, etc.) that are capable of sensing the physical world and sending their data observations (which we refer to as IoT data) to IoT applications. These IoT applications use collected IoT data to make decisions, enabling the development of smart IoT services and products that solve problems that were previously very difficult to solve. Deploying, managing, and maintaining IoT devices for the exclusive use of an IoT application is inefficient and involves significant costs, time, and effort that often outweighs the benefits [1]. Enabling the global sharing of third-party IoT devices that have been deployed, maintained, and owned by different parties (which we refer to as IoT device providers, or simply providers) significantly reduces the cost, timeframe, and effort of realizing IoT applications. Although this development can springboard cheaper and faster developments of novel smart IoT services and products [2], sharing third-party IoT devices (termed simply IoT devices for the remains of the paper) that are deployed and managed by different providers remains unrealized due to a lack of techniques to support global (global refers to the ability to share IoT devices deployed across the globe without the need to own them (similar to the Internet)) sharing (i.e., discovering, using, and paying) for IoT devices.

To illustrate the need for sharing IoT devices globally, we provide the following climate change example. The negative impact of climate change on agriculture can be mitigated by using IoT devices that provide the information needed to determine how various plants perform under changing environmental conditions across the world [3]; however, the procurement, deployment, and maintenance of IoT devices that are needed to monitor micro-climate, soil humidity, solar radiation, and crop performance are difficult to implement at this scale and incredibly expensive. Alternatively, using existing IoT devices, that have been deployed by farmers and agribusinesses for their own purposes to collect the data needed for climate change mitigation, can minimize the effort, cost, and timeframe for responding to the effects of climate change. This can be enabled through global IoT device sharing.

To achieve the global sharing of IoT devices, there is a need to provide the following objectives:

1. Discovering IoT Devices: This objective allows IoT applications to discover the IoT devices that they need from devices that have been made available by various providers. In our earlier example, this would enable an IoT application for climate change mitigation to discover devices already deployed IoT by farmers and agribusinesses, which can be potentially used to take mitigation actions that reduce the impact of climate change in agriculture.
2. Using IoT Devices: This objective allows IoT applications to use the IoT data of already deployed IoT devices instead of procuring, deploying, and maintaining their own devices. In our earlier climate change mitigation example, this would minimize the effort, cost, and timeframe of determining the impact of climate change on crops, and would enable mitigation actions to be taken, e.g., increase irrigation, use crops that require less water or tolerate worsening environmental conditions.
3. Paying IoT Devices: This objective allows IoT applications to share the cost of procuring, deploying, and maintaining IoT devices by paying the providers for their use (i.e., in our climate mitigation example, these would be farmers, agribusinesses, or other third parties). This is similar to the “pay-as-you-go” model seen in cloud computing [4].

While meeting the above objectives, it is equally important to address the following challenges for the global sharing of IoT devices via the Internet.

1. The Internet is an open system (i.e., no specific individuals and/or organizations own it or control it [5]). The Internet provides the infrastructure to allow for interconnection with devices owned by specific entities. However, the infrastructure itself is not owned by a specific entity; instead, thousands of collective people and organizations (entities) across the globe own the Internet [6]. Hence, we must develop a global sharing of IoT devices that are not owned or controlled by specific entities. We use the terms IoT-ownership or IoT-owned. The IoT-owned sharing of devices encourages IoT devices to be owned by different providers, but the infrastructure is not owned by any specific entity;
2. The Internet can be scaled up by supporting an ever-increasing number of users. Similarly, the global sharing of IoT devices requires the ability to scale up for potentially billions of IoT devices and IoT applications, while new ones are continuously added; we term this scalability;
3. The Internet allows computers that are manufactured by different companies and use different operating systems to communicate and share heterogeneous data that makes it interoperable [7]. Therefore, the global sharing of IoT devices requires support for IoT devices that have diverse sensing capabilities, heterogeneous hardware, different protocols, are manufactured by a variety of vendors, and generate heterogeneous data. In our climate change mitigation example, some available IoT devices may provide ground temperature measurements in Celsius, and other IoT devices may provide only combined ambient temperature and humidity measurements, while an application that uses such IoT data needs to construct a soil temperature map in Fahrenheit for temperatures collected from around the world during the summer months. Basic interoperability is achieved when at a minimum the descriptions of IoT data that are generated by available IoT devices are shared with the IoT applications when they are in the stage of discovering the IoT devices they need;
4. There is a requirement to develop the global sharing of IoT devices that is comprehensive. That means it should support all or most aspects of sharing (more specifically discovering) IoT devices. In our climate change mitigation example, one IoT application may look to discover IoT devices based on their data type, another may look for the geographical area of IoT devices, and another may look for the cost of using IoT devices and payment options, while another one looks for a way to use these IoT devices. Therefore, comprehensive discovery is needed to support the global sharing of IoT devices and to support all or most IoT application needs.

The main contributions of this paper are as follows:

- Providing a review of the most relevant articles and research outcomes supporting the sharing of IoT devices. This is achieved by following a SLNA-based methodology (Systematic Literature Citation Network Analysis);
- Proposing a taxonomy of challenges that helps classify and review related work in the area of sharing IoT devices and outlines related work gaps.
- Reviewing the related work for sharing IoT devices classified by the proposed taxonomy;
- Providing related work gap analysis and directions for novel research that can help other researchers advance the development of global sharing IoT devices.

To the best of our knowledge, this paper presents the first survey of techniques for discovering, using, and paying for third-party IoT devices. The survey area is important due to the benefits of reducing the cost, time, and effort of creating new IoT applications by discovering, using, and paying for third-party IoT sensors instead of procuring, deploying, and maintaining new sensors. The remainder of this paper is organized as follows: In Section 2, we discuss the survey methodology we used to collect and filter papers that are relevant to sharing IoT devices. In Section 3, we present a taxonomy of the challenges in devising the global sharing of IoT devices. Section 4 presents the existing techniques that can contribute to the research and development of the global sharing of IoT devices, while Section 4 provides a related work gap analysis and presents directions for novel research toward the developing the global sharing of IoT devices. Section 5 concludes the paper.

## 2. Survey Methodology

**Figure 1:**
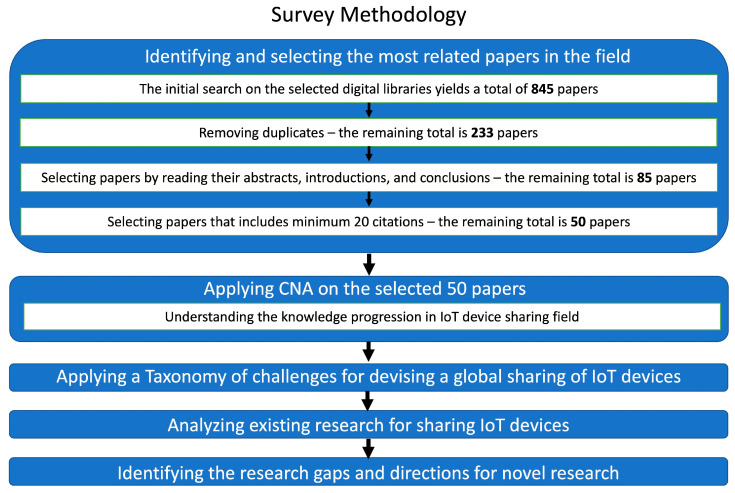
Structure of our survey methodology which is inspired by Systematic Literature Network Analysis (SLNA).

To identify and review the most relevant and cited related work on the sharing of IoT devices, we present our methodology, which is inspired by a Systematic Literature Network Analysis (SLNA). It combines a Systematic Literature Review (SLR) and Citation Network Analyses (CNAs) [8]. The SLR is used to identify ideas and select terms for the initial selection of the most relevant papers in the field. The CNA distinguishes a backbone in a citation network, which assists researchers in recognizing how the frame of knowledge has progressed over time [8]. In Section 2.1, we outline the methodology used for identifying and selecting the most relevant papers. Then, in Section 2.2, we present how we used the CNA [9] to analyze the knowledge progression in the field of study [10]. Figure 1 shows the structure of our survey methodology.

### 2.1. Identifying and Selecting the Most Relevant Papers

To identify the most relevant papers on sharing IoT devices, we used a combination of the terms listed in Table 1 to search the digital libraries of Google Scholar, Microsoft Academic, IEEE Xplore, Scopus, Emerald, and ScienceDirect. Please note that the search terms and expressions in Table 1 are the global IoT device sharing objectives and challenges we discussed in Section 1. Other aspects (e.g., power consumption) that are important for sharing IoT devices are out of scope of this paper. To search these digital libraries, we used the search strings in Table 1, combining multiple terms, to keep the search focused on sharing IoT devices. The “*” sign in Table 1 refers to a search for various forms of the word. The search on the selected digital libraries of the period between 2010 to 2023 yielded a total of 845 peer-reviewed publications (with duplicates, including journals, conference proceedings, and books). Peer-reviewed publications were used as they provide better quality than non-peer-reviewed ones. We selected the period between 2010 and 2023 as the modern IoT research and applications era started in mid-2010 [11]. After removing duplicates, we reduced the total number of related articles to 233. Next, we filtered this collection by reading the abstract, introduction, and conclusions of each paper. This stage reduced the number of papers to 85. We then filtered the papers based on their number of citations, as this reflects each paper’s contribution to and influence on this field of study [8]. To apply a fair citation filtration of recent papers, papers published in the last two years were excluded from the citation filtration, papers published in the last five years had to have at least five citations to be included in the review, and papers published more than five years ago had to have at least 20 citations to be included in the review. This stage removed 35 papers, reducing the total number of papers to 50. We included these 50 papers in the Citation Network Analysis that is discussed next in Section 2.2.

### 2.2. Applying CNA to Analyze Knowledge Progression

**Figure 2:**
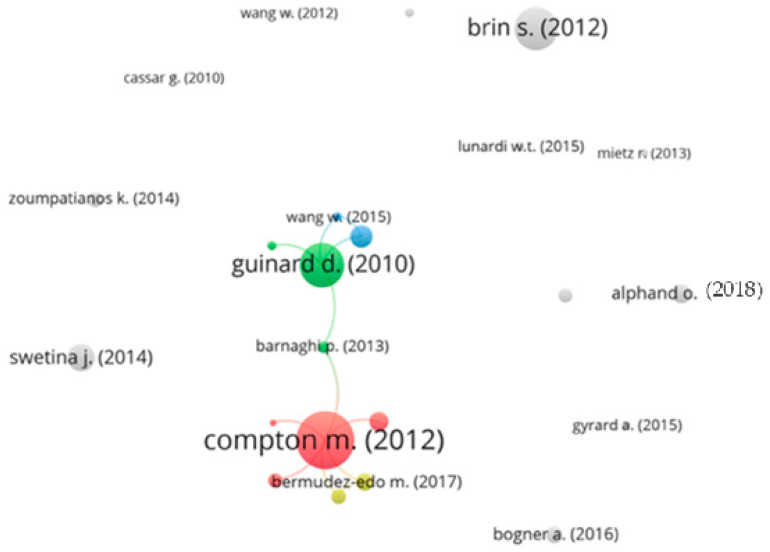
Citation analysis of the selected papers.

In this section, we applied CNA to the 50 selected papers. CNA reveals knowledge progression over time [8]. To identify the citation sources for each paper in the related work collection, we used Scopus to source the citation information (e.g., title, authors, year, and references). Next, we used VOSviewer [12], which is a specialized tool for visualizing a citation network, to perform a citation analysis of the selected papers. The results of the citation analysis of these highly cited papers is shown in Figure 2.

Note that the VOSviewer software [12] has some limitations, which prevented us from comparing all of the selected papers. These limitations include supporting a limited number of digital libraries (e.g., Google Scholar is not supported) and having limited citation formats. For those reasons, the works of Le-Phuoc et al. [13] and Harris et al. [14] were not included in the CNA. In Figure 2, papers are represented by circles; the size of these circles represents the number of citations of each paper. The lines between the circles represent the citation links between papers. Two major clusters are connected through Barnaghi et al. [15], as can be seen in Figure 2. These clusters represent the well-established field of IoT device discovery (majority of papers) and IoT devices use (minority of papers). This cluster was started in 2010 through papers on (1) the basics of describing IoT devices to discover them, and (2) techniques to integrate and use IoT devices and their data. We can clearly see that the work produced by Compton et al. [16] is the most cited in the field of sharing IoT devices, as it helped to establish the base of semantic descriptions of IoT devices. Compton et al. [16], along with Guinard et al. [17], represent the backbone of the discovery and use of IoT devices.

Other papers (coloured grey) have no citation link with any other paper in this analysis, as can be seen in Figure 2. These papers mainly provide techniques for querying, indexing, and integrating IoT devices without following the bases established by other researchers.

The CNA analysis helped us understand that the knowledge progression in the field of sharing IoT devices focuses on the (1) semantic discovery of IoT devices, which is enabled by describing IoT devices semantically to support semantic queries, and (2) integration of IoT devices to use their data.

### 2.3. A Taxonomy of Challenges for Devising the Global Sharing of IoT Devices

In this section, we propose a taxonomy of challenges that we use to review and classify work related to sharing IoT devices in order to devise the global sharing of IoT devices. Table 2 presents the proposed taxonomy.

This section may be divided by subheadings. It should provide a concise and precise description of the experimental results, their interpretation, as well as the experimental conclusions that can be drawn.

1. Describing IoT devices and their data: This category includes existing techniques (e.g., ontologies, data models, and schemas) that have been proposed to describe IoT devices and their data. IoT device and data descriptions are standards set of information about the software, hardware, system, and measurement capabilities, which can be used to describe sensors on IoT devices and their data (e.g., type, name, data unit, and location). Describing IoT devices is the key to discovering them and sharing them because it provides the necessary information that allows IoT applications to find the required IoT devices and IoT data. Simple descriptions of IoT devices prevent IoT applications from finding the required IoT devices and IoT data. For example, assume that a basic value name pairs technique describes IoT devices like “Type”: “temperature”, “location”: ”Australia”, “name”: “lab temperature IoT device”. This basic description does not provide enough information to differentiate a temperature IoT device from an “ambient air temperature IoT device” or a “system/machine temperature IoT device”. This prevents IoT applications from finding the required IoT devices. To help devise a global, IoT-owned, scalable, and interoperable sharing of IoT devices, a technique for describing IoT devices and their data should address the following challenges: (1) describe heterogeneous IoT devices and their data and support interoperability, and (2) provide comprehensive information to describe all possible aspects, including ownership of IoT devices to pay owners (providers) for using their IoT devices, the cost of using IoT devices to support paying for IoT devices, and the integration IoT devices detail to support IoT device use.
2. Registration of IoT Devices: This category includes existing techniques that have been proposed to manage and store the description of IoT devices to make them discoverable by IoT applications. To help devise a global, IoT-owned, scalable, and interoperable sharing of IoT devices, a technique for IoT device registration should address the following challenges: (1) scale up to the large and ever-increasing number of IoT devices, and (2) allow any device across the globe to be registered (i.e., IoT-owned) without owning the description of the IoT device.
3. Querying of IoT Devices: To discover IoT devices, IoT applications are required to query IoT devices and retrieve their descriptions based on IoT application needs. To help devise a global, IoT-owned, scalable, and interoperable sharing of IoT devices, a technique for IoT device query should address the following challenges: (1) scalability to query the large and ever-increasing number of IoT devices, (2) ability to query heterogeneous IoT devices and their data to support interoperability, (3) IoT-ownership to query IoT devices globally without control from any entity, and (4) comprehensiveness to query all or most possible aspects for finding IoT devices that match all or most IoT application needs. However, querying a large number of IoT devices and data descriptions (potentially billions) will increase the time required to find IoT devices and may impact the scalability of the global sharing of IoT devices. Therefore, there is a need for IoT device indexing that indexes IoT devices and data descriptions to support fast querying and, therefore, support the scalability of the global sharing of IoT devices. To help devise a global, IoT-owned, scalable, and interoperable sharing of IoT devices, a technique for IoT device indexing should address the following challenges: (1) be scaled to index the large and ever-increasing number of IoT devices, (2) be able to index heterogeneous IoT devices and their data to support interoperability, and (3) be IoT-owned to index IoT devices globally without control from any entity.
4. Integrating IoT Devices: To use IoT devices, there is a need for a technique (e.g., protocol and middleware) that can integrate IoT devices with IoT applications by establishing a communication channel between them, allowing IoT applications to use IoT data generated by IoT devices. To help devise a global, IoT-owned, scalable, and interoperable sharing of IoT devices, a technique for IoT device integration needs to address the following challenges: (1) support the integration of heterogeneous IoT devices (i.e., use different software, vendors, and integration protocols) and their data to support interoperability, (2) scale up to integrate a large number of heterogeneous IoT devices and their data, and (3) be IoT-owned to integrate IoT devices and their data without control from any entity.
5. Paying IoT Devices: This category includes existing techniques that have been proposed to enable a pay-as-you-go model to pay providers for sharing their IoT devices and data. To enable paying IoT devices, there is a need for a pay-as-you-go model that allows IoT applications to (1) pay for IoT device providers as long as they are using their IoT devices and data, and (2) be compensated in case the IoT data are not fully received due to any issue with the IoT device, such as low battery. To help devise a global, IoT-owned, scalable, and interoperable sharing of IoT devices, a technique for pay-as-you-go IoT device payment needs to (1) be scalable to manage payment and compensation transactions for a large and ever-increasing number of IoT devices, (2) support heterogeneous payment options such as cryptocurrencies, PayPal, and bank cards, and (3) support IoT-owned payment, i.e., allowing any IoT application to pay any IoT device globally with any payment option without any control from any entity.

## 3. Related Work for Sharing IoT Devices

This section provides a review of the existing techniques that contribute to the development of the global sharing of IoT devices. The existing techniques are classified based on our taxonomy provided in Section 3 and are reviewed based on their ability to address the challenges that are represented in the taxonomy.

### 3.1. Related Research for Describing IoT Devices and Their Data

Describing IoT devices and their data can be achieved via using techniques that use an ontology, a data model, or a schema. Most relevant papers use ontologies to describe IoT devices and their data. The most commonly used ontologies in the related work are Semantic Sensor Network (SSN) [16] and Sensor Observation Sampling Actuator (SOSA) [18], which are World Wide Web Consortium (W3C) recommendations that were developed by the W3C semantic sensor network incubator group (SSN-XG) [16,19,20]. SSN/SOSA are semantic-based ontologies that present a standard machine-readable semi-autonomous or even autonomous system to find, assemble, process, analyze, and proceed on IoT devices. The SSN ontology can be seen from four main perspectives, which are (1) the sensor perspective, which focuses on what senses, how it senses, and what is sensed; (2) the observation perspective, which focuses on observation data and related IoT device descriptions; (3) the system perspective, which focuses on deployments and systems that IoT devices belong to; and (4) the feature and property perspective, which focuses on what senses a particular property or what observations have been made about a property. SSN/SOSA can describe heterogeneous IoT devices and their data. Many researchers have used SSN/SOSA ontologies (modified or as is) in their work, such as Le-Phuoc et al. [13], Perera et al. [21], Kamilaris et al. [22], and Wang et al. [23].

The Machine-to-Machine Measurements (M3) ontology [24] has been proposed to solve some of the limitations of the existing standards (e.g., SSN [16], oneM2M [25], and ETSI M2M [26]). M3 is a semantic-based ontology, and it can describe heterogeneous IoT devices and their data. Also, it provides a unified description for sensors, data, and measurements, but it is limited to measurement name, device name, and unit of measurement. The authors have provided a link for the complete unified description, but it is not reachable. Other researchers who have used the M3 ontology for their work, such as the authors of [27,28], have modified the M3 ontology to fit their objectives for providing a unified semantic search engine for smart cities and the IoT. MyOntoSens [29] provides a semantic open data model for IoT devices and their data. The data model is designed to deal with the heterogeneity of IoT devices and their data. Although this data model was proposed mainly for the Wireless Sensor Networks (WSNs) domain, the model was designed based on other ontologies such as SSN [16], which makes it suitable for the IoT as well.

IoT-Lite [20] provides an instantiation of SSN [16] that allows for the interoperability and discovery of IoT devices by using lightweight semantics. IoT-Lite focuses on three key concepts, which are objects, devices, and services. It provides fast processing times for semantic queries by reducing the complexity of concepts and relationships. Barnaghi et al. [15] have proposed data modelling and annotation to describe the observation and measurement of streaming data. The data stream schema includes value, time, location, type, and unit of IoT data. Although this semantic model describes data observation and measurements, it does not cover describing IoT devices. Bharti et al. [30] have proposed a semantic resource (IoT device) ontology that describes a physical device and its characteristics. This ontology has four main concepts, which are (1) physical characteristics, including IDs, energy, and processing time; (2) working features, including accuracy, latency, and measurement; (3) deployment, including geographical location; and (4) interests, including device property. The Open Geospatial Consortium’s (OGCs) Sensor Web Enablement (SWE) group have proposed the Sensor Model Language (SensorML) for describing and discovering IoT devices [31]. The ontologies/models proposed in [15,20,30,31] cannot deal with heterogeneous devices and their data.

Bogner et al. [32] use their own schema to describe internet-connected objects (i.e., cars, bikes, and scooters) via IoT devices. This schema describes the cost of sharing these objects via IoT devices. However, the description focuses on the object, not on IoT devices.

Based on this review of the related work describing IoT devices and their data, only the ontologies/models proposed in [16,18,24,29] have the ability to describe heterogeneous IoT devices and their data. However, no ontologies/models in the related work provide the ability to support interoperability, i.e., provide a description of IoT devices and their data that allows IoT applications to understand heterogeneous IoT devices and their data automatically. To illustrate, each IoT device sends data in different sequences and formats. For example, consider two weather station IoT devices sending data to an IoT application: the first IoT device sends data in the following sequence and format (sensor 1 sends a value and its unit; sensor 2 sends a value only; and sensor 3 sends a value, its unit, and a time stamp of captured value); the second IoT device sends data in the following sequence and format (sensor 2 sends a value only; sensor 3 sends a value and the duty-cycle of capturing values; and sensor 1 sends a value and a unit). In this case, the IoT application does not understand the received data from these weather station IoT devices. These techniques need a standard or unified measurement that describes the syntax and format of the IoT data they send. While Barnaghi et al. [15] and Gyrard et al. [24] have proposed a schema towards unified sensor measurements, their schema does not consider the structure of an IoT device that includes several sensors, which requires other attributes to differentiate sensors in one IoT device. Also, none of the related work provides a comprehensive description of IoT devices and their data as can be seen in Table 3. For example, none support describing (1) the cost of using IoT devices, (2) the details of integrating IoT devices, and (3) the ownership of IoT devices.

### 3.2. Related Research for IoT Devices Registration

The registration of IoT devices involves managing and storing the descriptions of IoT devices.

Perera et al. [21] have proposed registering IoT devices in a MySQL database [33]. The authors have claimed that this database is limited to joining 61 tables only, which later on impacts querying IoT device descriptions as full IoT device description properties cannot be retrieved. To solve this issue, the authors have suggested using different storage methods or applying several queries at the same time. The authors have focused on evaluating the scalability of querying IoT devices registered in the MySQL database without consideration of evaluating the scalability of storing IoT device descriptions in the database. Lunardi et al. [34] have managed and stored IoT device descriptions using a PostgreSQL database [35]. The authors have evaluated their work by registering up to 1 million devices (i.e., IoT devices) without any significant issues with the database.

Barnaghi et al. [15] have proposed using distributed repositories that support managing and storing semantic IoT device descriptions. The authors have used a clustering algorithm to distribute the IoT device descriptions among diverse clusters in order to enable faster queries for retrieving the IoT device descriptions. However, their evaluation does not cover the scalability of managing and storing data streams. Wang et al. [36] have proposed managing and storing IoT device descriptions using semantic-based distributed gateways in order to provide scalable queries and support a range of different queries. The authors have evaluated managing and storing up to 10,000 IoT device descriptions. Paganelli and Parlanti [37] have proposed distributed repositories to manage and store IoT device descriptions. However, their evaluation does not cover the scalability of registering IoT devices.

GeoCENS [38] have used a hybrid peer-to-peer network for registering IoT devices and providing IoT device discovery. Their network consists of two major types of sensor web servers: (1) powerful servers that are maintained by large organizations, such as NASA, which are less likely to disappear or provide degraded services (i.e., very low volatility), and (2) less powerful servers that are managed by people or small organizations that are more likely to join and leave the network (i.e., high volatility). Their work has been used by different projects, which means that the GeoCENS network has been successfully deployed, but their paper has no evaluation of the scalability of managing and storing large numbers of IoT device descriptions. Although the storage of IoT device descriptions is considered an IoT-owned storage as no one specifically owns or controls it, the registration technique is generally controlled by the GeoCENS solution, which is not IoT-owned.

The authors of IOT Chain [39] proposed using a peer-to-peer blockchain to register IoT devices. It has been proposed as a decentralized consensus mechanism to manage IoT device descriptions, and it uses a private Ethereum blockchain to store IoT device descriptions. From an evaluation of the solution, the authors have estimated that the blockchain-based IoT could be influential and could produce a significant revolution. Wen et al. [40] have proposed a Blockchain-based Supply Chain System (BSCS) to provide a traceable and trustworthy mechanism to register IoT devices and store their data. Rahman et al. [41] have proposed using a permissioned Ethereum blockchain to provide a secure and privacy-assured data storage method for medical data. This system aims to (1) store the patients’ information along with data provided by related medical IoT devices, such as human body temperature; and (2) share the data with related stakeholders via a permissioned smart contract for access control. IOT Chain, BSCS, and Rahman et al. [41] use IoT-owned registration techniques for managing and storing IoT device descriptions and data. However, IOT Chain and BSCS have no evaluation of the scalability of managing and storing a large number of IoT devices’ metadata. Rahman et al. [41] have evaluated a blockchain with up to 5000 transactions and two mining nodes. However, this evaluation does not show the scalability of the system when dealing with a large number of IoT devices and transactions. Also, data storage is expected to grow exponentially as it stores the data of medical IoT devices and not just their descriptions and the patients’ information. Bogner et al. [32] have proposed using an Ethereum-based decentralized application (DAPP) for managing and storing descriptions of the objects (e.g., cars, bikes, and apartments, which are like IoT devices in our perspective) in an IoT-owned decentralized ledger. The storage of IoT device descriptions is considered to be IoT-owned, but managing IoT device descriptions is not. Also, the Ethereum blockchain is not scalable enough to register a large and ever-increasing number of IoT devices.

HCL-BaFoG [42] is a fog computing architecture that collects IoT devices’ data and stores them. HCL-BaFoG utilizes permissioned blockchain functionalities to provide secure IoT data sharing for clients (IoT applications). Although HCL-BaFoG stores IoT data and shares it, it does not have IoT device registration functionality. Given the permissioned architecture of HCL-BaFoG, it cannot be scaled up for IoT.

Based on this review of related work, most do not support a scalable and heterogenous registration of IoT devices, while Wang et al. [36] and Lunardi et al. [34] provide evaluations that show the registering of up to 1,000,000 IoT devices as can be seen in Table 4. With the increasing number of IoT deployments, there is a significant scope to expand these evaluations to billions of IoT devices. Also, the related work provides very limited support for IoT-owned registration. IOT Chain [39] and Wen et al. [40] have proposed a decentralized consensus mechanism that manages IoT device descriptions without control from any specific entity. However, it does not support storing the descriptions of heterogeneous IoT devices and it is not scalable for registering a large number of IoT devices.

### 3.3. Related Research for Querying IoT Devices

Querying IoT devices involves using attributes such as Data Type: Temperature and Location: Melbourne City to find IoT devices (more specifically, finding IoT devices and data descriptions) that match an IoT application’s needs. An example of such a query can be detecting the battery level of electric cars located less than 5 km from the Melbourne city center; the IoT device query technique should find the IoT device description that has a type: battery level, system: electric car, and geospatial: circular area with a radius of 5 km from Melbourne city center.

Google search [43] uses a scalable hypertextual search technique that can provide high-precision results. However, this technique is not designed for querying IoT device descriptions as they are much more complicated than websites.

Simurgh [44] have used a two-phase query technique based on the syntax of IoT device descriptions. The first phase is responsible for finding the matching IoT devices via a syntax-based query. The second phase is responsible for finding the required Application Program Interface (API) for the selected IoT devices in order to integrate them. Lunardi et al. [34] have proposed an IoT device query technique that can check synonyms and find other words with similar meanings and stop words that are not relevant to the query. Also, this technique provides a geographical interface for IoT applications to help them select the best match for IoT devices. GeoCENS search [38] have proposed using a hybrid peer-to-peer network (See Section 3.2 to allow for the distributed query of IoT devices. This technique allows finding even non-GeoCENS OGC web services (OWSs) (i.e., IoT devices) that are not registered in the hybrid peer-to-peer network. GeoCENS utilizes SOS [45] and SensorML [45] to enable the querying of IoT devices. This technique is scalable for a large number of stored IoT devices. The IoT device querying proposed by [34,38,44] uses matching keywords techniques, which lack flexibility for answering IoT device queries [17].

Simple Protocol and RDF Query Language (SPARQL) [14] is an RDF query language that has the ability to manipulate and retrieve RDF data. SPARQL supports a variety of queries and can return accurate results that match the IoT application’s needs. Also, SPARQL is scalable for querying a large number of registered IoT devices. There are several papers that have proposed using SPARQL as a query technique, such as Perera et al. [21], Wang et al. [34], Gyrard and Serrano [27], Barnaghi et al. [15], Le-Phuoc et al. [13], Mietz et al. [41], and Kamilaris et al. [22].

Based on the above review, most techniques in the related work have the ability to query heterogeneous IoT devices and their data. However, none of the related work supports comprehensive queries, mainly due to the lack of ontology describing IoT devices as IoT device query techniques cannot search for information that has not been described. The IoT device query techniques proposed in [34,38,43,44] are not IoT-owned as they are centralized and/or controlled by specific individuals or organizations. The current implementations of SPARQL [14] are on centrally owned infrastructure. Also, according to the review of related work, the IoT device query techniques proposed in [34,38,44] did not provide evidence for their scalability. Lunardi et al. [34] provide an evaluation for querying up to 1,000,000 IoT devices. Wang et al. [36] and Perera et al. [21] have experimented for up to 500,000 IoT devices to evaluate the querying of IoT devices using SPARQL as can be seen in Table 5. These evaluations were based on centralized databases and not on an IoT-owned infrastructure.

As we explained earlier in Section 3, there is a need for indexing IoT devices and data descriptions to support fast queries and, therefore, support the scalability of IoT device query techniques and the global sharing of IoT devices.

Wang et al. [36] have proposed using a geospatial indexing technique to reduce the search space for a faster IoT device query. They aimed to divide IoT devices based on geographic area, where the IoT device discovery was divided into distributed gateways and each gateway stored the description of IoT devices that are connected to it. Linked Sensor Middleware (LSM) [13] uses a spatial indexer for indexing IoT devices. LMS provides a basic indexing technique based on the type and location of IoT devices [21]. These techniques are limited to spatial indexing and cannot index IoT devices based on other properties (e.g., IoT device type or data units). The experiment conducted by Wang et al. [36] was limited to one small geographical area and a maximum of 1000 sensor services.

Perera et al. [21] have proposed using a Comparative-Priority-based Weighted Index (CPWI) technique for indexing IoT devices based on an IoT application’s needs. This technique indexes IoT devices during query time by calculating the similarity between the IoT application’s needs and the stored IoT device and data descriptions via a priority-based, weighted Euclidean distance in a multi-dimensional space mechanism. They have also applied heuristic filtering and a filtering-based relational expression to minimize the total amount of IoT devices and data descriptions that are required to be processed. This technique is able to index heterogeneous IoT devices. Perera et al. [21] have conducted an experiment to index up to 1,000,000 IoT devices with an indexing time that did not exceed 1000 ms.

Cassar et al. [46] have proposed using Probabilistic Latent Semantic Analysis (PLSA) and Latent Dirichlet Allocation (LDA) for indexing IoT devices based on a comparison between the IoT application’s query and the IoT device and data descriptions. There was no experiment to show the scalability of this technique.

Liang and Huang [38] have used a space-filling technique to index the sensor data (i.e., IoT data). Also, Tree-based (e.g., LOST-Tree) techniques were used to index IoT data by using spatial information. Barnaghi et al. [15] have used geospatial and temporal information to index IoT data. They have used Geohash tagging to provide geospatial information. Singular Value Decomposition (SVD) has also been used to reduce the dimensionality of Geohash vector data before applying a k-means clustering algorithm to allocate IoT data among repositories. Zoumpatianos et al. [47] have claimed that the main bottleneck for IoT device discovery is the indexing technique. Therefore, they have proposed shifting part of the time that is required for indexing IoT data from the IoT device registration to the IoT device query to provide faster IoT device discovery. The authors did this by indexing the parts of IoT data that are related to the IoT application’s query during query time. The authors introduced a mechanism that produces a tree of iSAX [48], which represents each of the data series (i.e., IoT data); the real data remains in the raw files and is loaded once a matched query appears [47]. The techniques proposed by Zoumpatianos et al. [47], Barnaghi et al. [15], and Liang and Huang [38] are designed to index data streams, which are not fully applicable to indexing IoT devices. Further, their evaluation does not cover large amounts of data (i.e., a large number of IoT devices and data descriptions) to show the scalability of these techniques.

Based on the review of the related work, most do not support the scalable and heterogenous indexing of IoT devices, while Perera et al. [21] provide an evaluation of indexing up to 1,000,000 IoT devices. With the increasing number of IoT deployments, there is a significant scope to expand these evaluations into billions of IoT devices. Also, none of the related work provides IoT-owned indexing techniques for IoT devices, as can be seen in Table 6.

### 3.4. Related Research for Integrating IoT Devices

A common technique for integrating IoT devices is using a wrapper that can virtually integrate any IoT device [19]. A wrapper is a piece of code developed individually to integrate each IoT device. These wrappers are usually developed by the developers of the system/solution that offers IoT device integration, or by the provider of the IoT device. Several solutions/systems have used wrappers to integrate IoT devices, including LSM [13], OpenIoT [49], and (GSN) middleware [50].

Application Programming Interfaces (APIs), which are software that allow for IoT applications to interact with IoT devices [51], can provide an interface for deployed IoT devices to be integrated with IoT applications. One API can provide an interface for integrating several IoT devices. Several solutions/systems have used APIs to integrate IoT devices, including Simurgh [44] and Agri-IoT [22]. Although using a wrapper or API technique can virtually support the integration of any IoT device, it is not scalable due to the need to develop a wrapper or API for each IoT device or group of IoT devices. Also, IoT-ownership depends on the system/solution that uses these wrappers or APIs.

Other IoT device integration techniques are providing information like Uniform Resource Locators (URLs) for IoT applications and are expecting IoT applications to use their own IoT platform or middleware to integrate IoT devices. Paganelli and Parlanti [37] have proposed providing a Universal Resource Identifier (URI) for the repository that has the required information to integrate IoT devices. Wang et al. [36] have provided unique identifications for IoT devices, such as endpoints that allow for IoT applications to integrate with IoT devices. The scalability, IoT-ownership, and ability to support heterogeneous IoT devices depend on the IoT platform and/or the middleware used by IoT applications.

Simurgh [44] uses APIs and low-level programming libraries to integrate IoT devices and obtain their data. These APIs are developed by Simurgh developers, volunteer developers, or IoT device providers. Simurgh contains an API access management layer that provides the necessary information to manage the integration of IoT devices.

Perera et al. [52] have proposed a Context-Aware Sensor Configuration Model (CASCoM) to provide automation in the integration of IoT devices for IoT middleware, such as GSN [50]. CASCoM has the ability to integrate IoT devices without the need for a wrapper or API, and it is easy to use for non-IT experts. However, CASCoM needs all the information related to IoT devices and data processing components to be stored in a repository. Perera et al. [52] have conducted an experiment to integrate up to 10,000 IoT devices. The experiment shows that CASCoM can be up to 250 times faster (respectively) in comparison to some existing techniques, such as GSN.

Madureira et al. [53] have proposed the Internet of Things Protocol (IoTP), which supports IoT data aggregation from the network layer among heterogeneous IoT devices to improve interoperability. IoTP does not rely on routing protocols; thus, it is a more generic protocol and supports interoperability. IoTP requires another layer to manage the aggregation of IoT data. The authors have ran an experiment with a total of 50 IoT devices to evaluate IoTP by measuring several metrics including transmitted data, network efficiency, and average delay.

Based on the review of related work, all of the techniques except for [53] do not provide support for interoperable integration due to either the need for an API or wrapper for almost each different IoT device. The technique proposed in [53] partially supports interoperable integration by allowing data aggregation for heterogeneous IoT devices. However, it requires an extra layer to manage the integrated data. The related work lacks an application layer protocol that fully supports the integration of any IoT device with any IoT application. Also, the related work provides very limited support for the scalable integration of IoT devices. Perera et al. [52] have used up to 10,000 IoT devices, and Madureira et al. [53] have used up to 50 IoT devices to evaluate their integration techniques as can be seen in Table 7. Further, none of the related work provides any support for the IoT-owned integration of IoT devices.

### 3.5. Related Research for Paying IoT Devices

IoT device payment techniques involve supporting a pay-as-you-go model for sharing IoT devices in a method similar to cloud computing [4]. Such a model allows IoT applications to pay IoT device providers as long as they use their IoT devices.

Ether cryptocurrency [54] is proposed by several solutions, such as [32] as a payment technique to pay for using IoT devices. However, based on the performance of the Ethereum blockchain [54], it is not scalable for paying for a large number of IoT devices. Also, it does not support a pay-as-you-go-model.

There are other techniques that can be used to provide payment for IoT devices, such as PayPal [55] and cryptocurrencies like Bitcoin [56] and MIOTA [57]. However, there is no paper that proposes using PayPal [55] or Bitcoin [56] to support paying IoT devices. Also, PayPal [55], Bitcoin [56], and MIOTA [57] require development to support a pay-as-you-go model for using IoT devices.

Existing IoT device sharing solutions/marketplaces do not support a pay-as-you-go model for using IoT devices.

Tzianos et al. [58] provide preliminary work on supporting a payment mechanism for IoT device sharing by using the IOTA wallet [57] to enable IoT clients to purchase data packets. However, Tzianos et al. [58] did not provide any model or mechanisms to process payment, calculate required payment, nor support pay-as-you-go payment for IoT devices.

Özyilmaz et al. [59] support IoT device sharing via a blockchain-based marketplace. Özyilmaz et al. [59] have proposed using the Raiden network [60] off-chain payment channel to support micro-payments for IoT device providers. Rahman et al. [61] have proposed a blockchain-based marketplace for sharing IoT devices. Rahman et al. [61] use EOS permissioned blockchain [62] to support paying IoT devices for their data. However, Özyilmaz et al. [59] and Rahman et al. [61] did not provide information for a pay-as-you-go model. Also, they did not provide a mechanism to calculate the required payment.

All of the discussed solutions, including [54,56,57,58,59,61], do not fully support paying IoT devices through a pay-as-you-go model as can be seen in Table 8.

## 4. Gap Analysis and Directions for Novel Research

**Figure 3:**
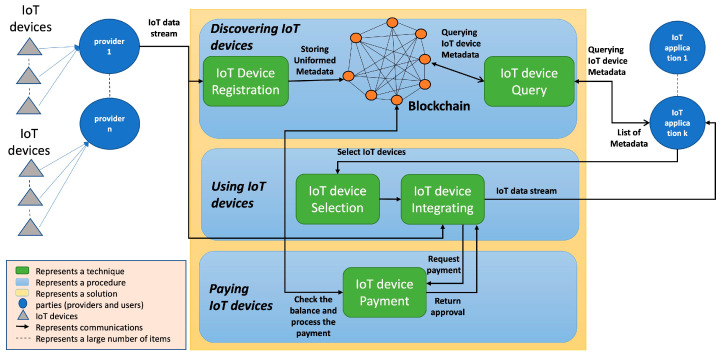
Our envisioned architecture for sharing IoT devices.

In the previous section, we reviewed the related work that can contribute to the development of IoT device sharing and enable its objectives, which are IoT device discovery, use, and pay. This review has helped us identify existing gaps in the related work IoT device sharing. In this section, we analyze the related work gaps that have hindered the development of global IoT device sharing. Also, we provide directions for novel research that help researchers advance the development of global IoT device sharing. From reviewing the related work in Section 4 and Table 3, Table 4, Table 5, Table 6, Table 7 and Table 8, we can see that most of the existing techniques do not address the challenges of achieving global IoT device sharing. From the reviewed techniques that are based on using, or on part of blockchain technology such as [30,37,38,39,40,55,56,57,59], we can say that blockchain technology is a possible approach to establishing global and IoT-owned IoT device sharing. Blockchains are a decentralized ledger that can allow for IoT applications to query IoT devices and data descriptions along with other needed information (e.g., endpoint) stored in them. Also, it can run layers or modules on top of it in a decentralized structure to support IoT device discovery, using and paying functionalities for IoT applications. Blockchain is not owned or controlled by individuals; it runs on globally distributed computers (i.e., nodes) and is controlled by a consensus. It can thus support the IoT-owned sharing of IoT devices. However, in our survey, there is no blockchain-based technique for indexing IoT devices. Also, the analyzed blockchain techniques did not provide proof of scalability for sharing a large number of IoT devices. Based on that, in this section, we describe the identified gaps that hindered the development of the global sharing of IoT devices and our vision to address these gaps. Figure 3 shows our envisioned architecture for IoT device sharing.

### 4.1. Describing of IoT Devices and Their Data

Existing techniques for describing IoT devices and data require further development to support the global sharing of IoT devices.

1. Semantic technology is highly relevant to describing IoT devices and their data as it provides the foundations for the development of sharing IoT device solutions. Semantic technology is proposed by the IoT community for providing IoT devices and data descriptions to enable interoperability [63]. The Semantic Sensor Network (SSN) ontology is the leading semantic description of IoT devices and their data and provides the basis of most of the well-known ontologies in this field. However, SSN is not comprehensive as it does not cover the ownership of IoT devices, location, and the measurement format of IoT data. Also, there is other information that is not covered by SSN, and this is required to support the integration and payment of IoT devices (e.g., payment ID for IoT devices, cost of using IoT device, Uniform Resource Identifier (URI), integration protocol, and special token for fetching IoT data from IoT devices). Thus, there is a need for a technique which describes IoT devices and their data and that (1) supports heterogeneous IoT devices and their data as well as heterogeneous IoT applications, and (2) provides a comprehensive description that facilitates all or most of the possible aspects, such as the description of payment and the integration of IoT devices. Early work on this area is published in [64]. The authors have proposed an SSN-based ontology that supports semantic queries, integration, and the payment of IoT devices. However, further development is required to fully support a comprehensive description and heterogeneous IoT application.
2. The problem with semantic description is that it requires both significant effort and considerable expertise in RDF-based ontology definitions and use [65]. We envision a machine learning-based description technique that is able to analyze the data stream of IoT devices to generate automatic semantic descriptions for IoT devices and their data. Early work in this area has been published in [66]. The author has proposed using k-fold cross-validation (CV) to train a novel metadata-assisted cascading ensemble classification framework (MACE) for annotating heterogeneous IoT data autonomously. However, this model does not generate the metadata needed for IoT devices.
3. Storing semantic information in a Blockchain requires a specialized blockchain that has an RDF triple store for the efficient query of IoT devices and data descriptions. Early work in this area is published in [1,2]. The authors have implemented an RDF triple store inside each of the distributed nodes of the blockchain in order to store semantic descriptions of IoT devices and data as triples. They have called this a semantic-based blockchain. However, further development is required to achieve an efficient triple store for a scalable semantic-based blockchain.

The techniques for describing IoT devices, automatically generating semantic metadata, and storing metadata in a blockchain can be called IoT device registration, as can be seen in Figure 1.

### 4.2. Querying IoT Devices

Existing techniques for querying and indexing IoT devices require further development to support the global sharing of IoT devices.

1. Querying IoT device techniques lacks scalability due to the complexity of semantic data and the large number of IoT devices and data descriptions. Therefore, we envision a distributed IoT device query technique that is deployed on each blockchain node. Each query can be processed by multiple nodes that each query part of the RDF-store concurrently to achieve a fast and scalable query technique. Further, deploying IoT device query techniques on the blockchain can make it IoT-owned as no entity owns or controls the query technique.
2. Semantic query languages, which are required to support semantic description such as SPARQL, are complex and require experts to write queries. Also, they are not comprehensive enough to query all or most of the possible aspects of IoT devices, mainly for as they lack a comprehensive ontology. Therefore, we envision the development of an easy-to-use and comprehensive IoT device query language that is able to provide the constructs needed to query IoT devices and support all or most IoT application needs.
3. Existing techniques for indexing IoT devices are not IoT-owned and lack scalability. We envision developing a novel blockchain-based indexing technique that provides fast access to semantically clustered descriptions of IoT devices and their data stored in a blockchain. Early work on this area is published in [67]. The authors have proposed providing (1) a lookup table index for semantic information among blocks and (2) a block-level recursive model index for blocks to improve the query efficiency. However, this work requires development to address the semantic description of IoT devices.

### 4.3. Integrating IoT Devices

Existing techniques for integrating IoT devices require further development to support the global sharing of IoT devices.

1. The integration of IoT device techniques lacks scalability because (1) they either rely on developing APIs or wrappers for almost each IoT device manually or require a set of information for each IoT device to create the wrapper automatically; and (2) there are no standards for developing these wrappers and APIs, as every provider has its own developed one. Therefore, we envision a machine learning-based integration technique that cooperates with the machine learning-based description technique discussed earlier in Section 4.1 to provide an automatic integration of IoT devices.
2. The integration of IoT device techniques lacks interoperability, which describes the ability for heterogeneous IoT applications to integrate the same IoT device and for one IoT application to integrate heterogeneous IoT devices. Therefore, we envision an application layer protocol that can cooperate with (1) existing Internet and IoT protocols, such as MQTT [68], CoAP [69], and HTTP [70]; (2) the machine learning-based description technique discussed earlier in Section 4.1; and (3) the machine learning-based integration technique discussed earlier in this section in order to (a) integrate heterogeneous IoT devices and collect their data, and (b) interpret collected data to formats and structures understandable by heterogeneous IoT applications.

### 4.4. Paying IoT Devices

Very few techniques in the related work support paying for shared IoT devices, and none of them support heterogeneous payments or a pay-as-you-go model. To support paying for globally shared IoT devices, we need to achieve the following: (1) identify the cost of reusing IoT devices per unit of time and/or per unit of IoT data (this part can be performed in IoT device and data descriptions as explained above in the first point); (2) provide a unique payment ID for IoT devices; (3) allow for IoT applications to query IoT devices based on their cost; (4) allow for IoT applications to pay for using IoT devices; (5) compensate IoT applications in case IoT data are not fully collected; and (6) support IoT-owned and heterogenous payments, such as cryptocurrencies, bankcards, and PayPal. Early work in this area is published in [64]. The authors have proposed an initial pay-as-you-go model that achieves some of the above requirements. However, the authors have not evaluated the model. Also, further development is required to (a) support the compensation of IoT applications in case IoT data are not fully shared, and (b) support heterogeneous payment.

### 4.5. Scalability

Using a blockchain-based structure for the global sharing of IoT devices supports IoT-ownership, but it may not be scalable to managing the data of a large number of IoT devices and IoT applications because it requires some time and effort to generate blocks (i.e., contain data). Therefore, there is a need for the following:

1. Limiting the size of information stored in the blockchain to ensure scalability for dealing with a large number of IoT devices. The authors in [71] have proposed a self-managed marketplace for sharing IoT devices using a blockchain. The authors limited the information stored in the blockchain by preventing IoT devices from storing their data in the blockchain and by only storing the IoT devices and data descriptions that are required for discovery, use, and pay. However, there is a need to reduce the size of this information by compressing it or storing part of it in off-chain storage.
2. Developing a new consensus mechanism for blockchain that ensures (1) scalability, (2) suitability to be run by IoT devices, and (3) the majority of nodes that generate blocks belong to the Internet of Things (e.g., IoT devices and gateways).

## 5. Conclusions

Global sharing of IoT devices is emerging as an important area, with a plethora of IoT application developments and deployments. The true value of IoT will be realized when it can be operated similarly to the internet, i.e., IoT device providers can share their IoT devices with consumers developing IoT applications and be rewarded for doing the same. In this paper, we have conducted an SLNA-based review of the related work regarding advances in techniques that support the development of the global sharing of IoT devices. We have proposed a taxonomy of challenges that helps to review and classify techniques for IoT device discovery, use, and pay. By reviewing the related work in IoT device sharing, we have found that existing techniques that enable discovering, using, and paying for IoT devices have come a long way but are still in the infancy of being able to support the global sharing of IoT devices. Existing techniques do not sufficiently cater to the challenges imposed by the IoT, such as IoT-ownership, scalability, and the heterogeneity of IoT devices and the data they generate. Our analysis leads to the identification of directions for novel research, including the development of a scalable and semantic blockchain-based marketplace for enabling automated data sharing from third-party IoT devices to IoT applications.

## Figures and Tables

**Table 1:** The keywords, databases, and strings for finding papers.

Search Terms	Databases	Search Strings
IoT, Internet of things, Internet of everything, IoT device, Device, Sensor, IoT sensor, IoT data, IoT service, IoT product, IoT resource, IoT source, Discovery, Use, Reuse, Cost-sharing, Pay, Trustworthy, Scalable, Variety, Heterogeneity, Comprehensive	Google scholar, Microsoft academic, IEEE Xplore, Scopus, Emerald, and ScienceDirect	(“sensor*” OR “device*” OR “IoT sensor*” OR “IoT device*” OR “IoT data*” OR “IoT service*” OR “IoT resource*” OR “IoT product*” OR “IoT source*”) AND (“discover*” OR “use*” OR “reuse*” “ OR “cost-share*” OR “pay*” AND (“IoT” OR “internet of things” OR “internet of everything”), (“scale*” OR “scale-up”, OR “trustworthy”, OR ”trust*”, OR “variety*” OR “heterogeneity” OR “comprehensive”) AND (“sensor*” OR “device*” OR “IoT sensor*” OR “IoT device*” OR “IoT data*” OR “IoT service*” OR “IoT resource*” OR “IoT product*” OR “IoT source*”) AND (“discover*” OR “use*” OR “reuse*” OR “cost-share*” OR “pay*”)

**Table 2:** Taxonomy of challenges of devising the global sharing of Internet of Things devices.

Taxonomy Categories		Challenges of Global Sharing of IoT Devices			
		Scalable	IoT-Owned	Interoperable	Comprehensive
Discovering IoT devices	Describing IoT devices and their data	N/A	N/A	Description of heterogenous IoT devices, their data, and supporting heterogenous IoT applications	Description of all or most aspects of IoT devices, including the cost/payment of using IoT devices and integration details
	Registering IoT devices	Registration of the large and ever-increasing number of IoT devices	Registration of any IoT device globally without owning the descriptions of the IoT device	Registration of heterogenous IoT devices	N/A
	Querying IoT devices	Query of the large and ever-increasing number of IoT devices	Query IoT devices globally without any control from any entity	Query heterogenous IoT devices and their data	Query IoT devices comprehensively including cost/payment and integration capabilities
	Indexing IoT devices	Index of the large and ever-increasing number of IoT devices	Index IoT devices without any control from any entity	Index heterogenous IoT devices	N/A
Using IoT devices	Integrating IoT devices	Integration of the large and ever-increasing number of IoT devices	Integrate IoT devices globally without any control from any entity	Integrate heterogenous IoT devices and obtain their data	N/A
Paying IoT devices		Payment of the large and ever-increasing number of IoT devices	Payment of IoT devices globally without any control from any entity	Dealing with heterogenous payment options to pay for using IoT devices	N/A

**Table 3:** The related research that addresses describing Internet of Things devices and data challenges.

Interoperable	Comprehensive	Fully Support Descriptions of IoT Devices and Their Data
Refs. [16,18,24,29] support heterogenous IoT devices, but show limited support for interoperability	None	None of the related work fully supports describing IoT devices and their data

**Table 4:** The related research that addresses Internet of Things device registration challenges.

Scalable	IoT-Owned	Interoperable	Full Support of Registering IoT Devices
Refs. [34,36] evaluate registration of up to 1,000,000 IoT devices	[39,40]	None	None of the related work fully supports registering IoT devices

**Table 5:** A summary of the review of related work for querying Internet of Things devices.

Scalable	IoT-Owned	Interoperable	Comprehensive	Full Support of Querying IoT Devices
Ref. [34] evaluates querying up to 1,000,000 IoT devices Refs. [21,36] evaluate querying up to 500,000 IoT devices	None	None	None	None of the related work fully supports querying IoT devices

**Table 6:** The related research that addresses indexing Internet of Things devices challenges.

Scalable	IoT-Owned	Interoperable	Full Support of Indexing IoT Devices
Ref. [21] evaluates indexing up to 1,000,000 IoT devices	None	None	None of the related work fully supports indexing IoT devices

**Table 7:** The related research that addresses integrating Internet of Things devices challenges.

Scalable	IoT-Owned	Interoperable	Fully Support Integrating of IoT Devices
Refs. [36,52] uses up to 10,000 IoT devices to evaluate their integration techniques Ref. [53] uses up to 50 IoT devices to evaluate the integration technique	None	None	None of the related works fully support integrating IoT devices

**Table 8:** A summary of the review of related work for paying Internet of Things devices.

Scalable	IoT-Owned	Interoperable	Fully Support Paying for IoT Devices
[57,58]	[54,56,57,58,59,61]	None	None of the related work fully supports paying for IoT devices nor supports pay-as-you-go
